# Disrupting the LC3 Interaction Region (LIR) Binding of Selective Autophagy Receptors Sensitizes AML Cell Lines to Cytarabine

**DOI:** 10.3389/fcell.2020.00208

**Published:** 2020-03-31

**Authors:** Mateusz Putyrski, Olesya Vakhrusheva, Florian Bonn, Suchithra Guntur, Andrew Vorobyov, Christian Brandts, Ivan Dikic, Andreas Ernst

**Affiliations:** ^1^Institute of Biochemistry II, Medical Faculty, Goethe-University, Frankfurt, Germany; ^2^Project Group Translational Medicine and Pharmacology, Fraunhofer Institute for Molecular Biology and Applied Ecology, Frankfurt, Germany; ^3^Department of Medicine, Hematology/Oncology, Goethe-University, Frankfurt, Germany; ^4^German Cancer Consortium and German Cancer Research Center, Heidelberg, Germany; ^5^University Cancer Center Frankfurt, Goethe-University, Frankfurt, Germany; ^6^Buchmann Institute for Molecular Life Sciences, Frankfurt, Germany

**Keywords:** phage display, selective autophagy receptor, LIR interaction, cytarabine, AML – acute myeloid leukemia, inhibitors, short linear motifs (SLiMs)

## Abstract

Short linear motifs (SLiMs) located in disordered regions of multidomain proteins are important for the organization of protein–protein interaction networks. By dynamic association with their binding partners, SLiMs enable assembly of multiprotein complexes, pivotal for the regulation of various aspects of cell biology in higher organisms. Despite their importance, there is a paucity of molecular tools to study SLiMs of endogenous proteins in live cells. LC3 interacting regions (LIRs), being quintessential for orchestrating diverse stages of autophagy, are a prominent example of SLiMs and mediate binding to the ubiquitin-like LC3/GABARAP family of proteins. The role of LIRs ranges from the posttranslational processing of their binding partners at early stages of autophagy to the binding of selective autophagy receptors (SARs) to the autophagosome. In order to generate tools to study LIRs in cells, we engineered high affinity binders of LIR motifs of three archetypical SARs: OPTN, p62, and NDP52. In an array of *in vitro* and cellular assays, the engineered binders were shown to have greatly improved affinity and specificity when compared with the endogenous LC3/GABARAP family of proteins, thus providing a unique possibility for modulating LIR interactions in living systems. We exploited these novel tools to study the impact of LIR inhibition on the fitness and the responsiveness to cytarabine treatment of THP-1 cells – a model for studying acute myeloid leukemia (AML). Our results demonstrate that inhibition of LIR of a single autophagy receptor is insufficient to sensitize the cells to cytarabine, while simultaneous inhibition of three LIR motifs in three distinct SARs reduces the IC_50_ of the chemotherapeutic.

## Introduction

Short linear interactions motifs (SLiMs) play a crucial role in the organization and assembly of intracellular signaling complexes and have been closely associated with the development of higher order organisms (Pawson and Nash, 2003; Beltrao and Serrano, 2007). These peptide motifs are predominantly embedded in disordered regions of otherwise large multidomain proteins and provide a structurally flexible binding interface for globular domains (Tompa et al., 2014; Van Roey et al., 2014). From an evolutionary point of view, disordered regions and their embedded binding motifs are advantageous as they allow the rapid development of new protein–protein interaction networks, thereby accelerating the evolution of complex signaling and regulatory pathways required for the orchestration of various intracellular processes in multicellular organisms. This insight becomes evident in the analysis of large scale interactomes, which have uncovered that disordered regions experience a higher rate of insertion and deletions (Mosca et al., 2012). Additionally, studies on the mutational flexibility of SLiMs and their binding partners demonstrated that interactions with altered selectivity required for the evolution of novel protein–protein networks can be easily established (Ernst et al., 2009; Teyra et al., 2019). Next to their evolutionary properties, the transient nature of SLiM interactions due to fast association/dissociation rates to their cognate binding protein is another characteristic of such interaction motifs (Sugase et al., 2007; Perkins et al., 2010). This transiency allows for an additional level of regulation, for example, by relying on multimerization of binding partners, i.e., binding avidity, or by posttranslational modification of binding site residues. Both effects serve to modulate the overall affinity of the SLiM to its globular binding partner and allow cells to dynamically adapt to changes in their surroundings. It is therefore astonishing that, despite their central role in the organization and regulation of signaling networks, no general approach exists to date to study the biology of SLiMs in live cells. Consequently, for the majority of conserved SLiMs it is unclear whether a disruption of their interactions can be exploited for a therapeutic benefit.

A prime example of a short linear binding motif in which the features of SLiMs are found is the LC3 interaction region (LIR), which binds to the LC3/GABARAP family of proteins. Canonical LIRs consist of four amino acids, with a consensus of ΘXXΓ, where X stands for any amino acid, Θ for Trp, Phe, Tyr and G for Leu, Val or Ile (Birgisdottir et al., 2013). Additionally, non-canonical LIR motives have been described, the most prominent example being, so called, CLIR (ILVV) of NDP52 (Von Muhlinen et al., 2013). As typical SLiMs, LIRs are evolutionary conserved interaction motifs that already occur in yeast and can be found in a large variety of different proteins in higher eukaryotes (Kirkin and Rogov, 2019). Additionally, posttranslational phosphorylation of residues in close proximity to LIR motifs has been shown to increase binding to their interaction partner (Wild et al., 2011). As globular binding partners for LIRs, the LC3/GABARAP family in humans consists of three members of the LC3 subfamily, LC3A, LC3B, and LC3C, and three members of the GABARAP subfamily, GABARAP, GABARAPL1, and GABARAPL2. All six members share the ubiquitin β-grasp fold, with an extend N-terminal helical part and have the ability to bind LIR peptides with μM affinity (Rogov et al., 2013; Sakurai et al., 2017; Wirth et al., 2019). A unique feature distinguishing the LC3/GABARAP family from other ubiquitin-like proteins is that, in addition to their LIR-binding properties, they are posttranslationally modified at their C-terminus with the lipid phosphatidylethanolamine (PE) (Kabeya et al., 2004). The LIR motif and their binding partners LC3/GABARAP proteins have a key role in the organization of macroautophagy (hence forth called autophagy) (Johansen and Lamark, 2011; Stolz et al., 2014).

Under basal and amino acid starvation conditions, autophagy is an indiscriminate process in which parts of the cytosol are sequestered by a double membrane structure (phagophore) into autophagosomes (Yoshimori, 2004). After fusion of the autophagosome with lysosomes, the sequestered material is broken down into its individual components by lysosomal enzymes (Lorincz and Juhasz, 2019). Next to the ubiquitin-proteasome system, autophagy is essential for cellular homeostasis and is required for the continuous recycling of the building blocks of intracellular proteins, carbohydrates, lipids and organelles. Under stress conditions, autophagy has cytoprotective functions and selective autophagy receptor (SARs) proteins specifically recognize damaged or unwanted material, such as damaged mitochondria (mitophagy), intracellular pathogens (xenophagy) or protein aggregates (aggrephagy) (Stolz et al., 2014). Amongst the best studied SARs is the group of p62/SQSTM1 like receptor proteins (SLRs) that is composed of the six proteins: sequestomsome 1 (SQSTM1 or p62), calcium-binding and coiled-coil domain-containing protein 2 (CALCOCO2 or nuclear dot protein 52: NDP52), optineurin (OPTN), next to BRCA1 gene 1 (NBR1), Tax1-binding protein 1 (TAX1BP1) and Toll-interacting protein (TOLLIP) (Kirkin and Rogov, 2019). These SLRs are composed of multiple domains with distinct functionalities that mediate binding to ubiquitinated cargo (UBAN, UBA, Zn, ZnF, CUE), lead to oligomerization (CC, PB-1) and, in case of NDP52, interact directly with intracellular membranes (SKICH domain) (Grubisha et al., 2010; Husnjak and Dikic, 2012; Von Muhlinen et al., 2012; Ciuffa et al., 2015; Li et al., 2018). In addition to folded segments, the SLRs contain LIR motifs in their unstructured regions, which mediate binding to LC3/GABARAP proteins. The latter are themselves tethered via their PE anchor in the membrane of the phagophore. Thus, SLRs link ubiquitinated cargo to autophagosomes. This modular separation into membrane bound LC3/GABARAP proteins and linear LIR motifs embedded in larger SARs allows for a great functional diversity of selective autophagy and numerous SARs have been described that facilitate the degradation of a wide range of different types of cargo by autophagy (Kirkin and Rogov, 2019).

In this work, we describe the protein engineering of specific inhibitors of LIRs and characterize their binding properties *in vitro* and their impact on the survival of THP-1 cells, a model cell line to study acute myeloid leukemia (AML). We predicated our protein engineering approach on previous work where we demonstrated that intracellular affinity reagents can be generated by introducing targeted mutations in the binding site of a naturally occurring binding partner (Ernst et al., 2013; Wiechmann et al., 2017, 2020). As scaffold to target LIRs, we chose the proteins LC3B and GATE-16 from the LC3/GABARAP family of LIR-binding proteins. Both proteins proved to be amenable to engineering and we could derive several variants with improved affinity relative to the corresponding wt interaction. In intracellular experiments, the variants bound to their cognate LIR motif in the context of the full-length receptors. Interestingly, experiments in THP-1 cells indicate that cells expressing LIR inhibitors have a growth disadvantage and become more sensitive to the treatment with the chemotherapeutic cytarabine (Stein and Tallman, 2016). Thus, our results provide evidence that interfering with LIR binding may have a therapeutic benefit in the treatment of AML.

## Results and Discussion

**FIGURE 1:**
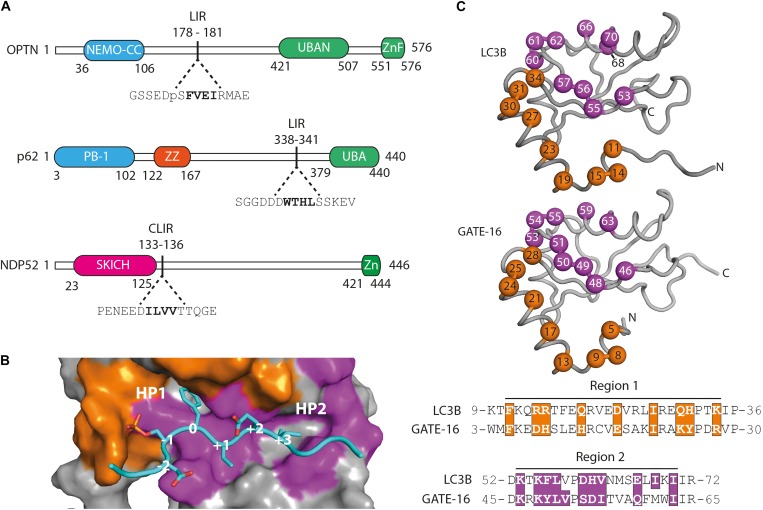
Functional domains in the targeted autophagy receptors and library design. **(A)** OPTN, p62 and NDP52 are multifunctional proteins that contain domains mediating multimerization (blue) and cargo recognition (green). In p62, the ZZ domain (orange) binds arginylated (Nt-R) substrates (Zhang et al., 2018). The SKICH domain (magenta) of NDP52 has been shown to bind to intracellular membranes (Von Muhlinen et al., 2012). The four amino acid linear LC3 interaction region (LIR) is shown in bold in its broader amino acid context. **(B)** Surface representation of the LIR binding site of LC3B in complex with a pS177 OPTN peptide (cyan). Region 1 (orange) and region 2 (purple) are shown. Hydrophobic binding pocket 1 (HP1) coordinates position 0 (Trp, Phe or Tyr) residues and HP 2 coordinates position +3 residues (Leu, Val or Ile) in canonical LIR peptides. **(C)** LC3B (pdb: 3VTU) and GATE-16 (pdb: 1EO6) library design. Surface exposed residues (spheres) in region 1 (orange) and region 2 (purple) that mediate binding to LIR motifs have been randomized and are shown below in the context of their aligned primary amino acid sequence. Amino acid positions of the boundaries of regions 1 and 2 are shown.

In order to examine whether selective autophagy can be modulated by disrupting LIR interactions, we have generated affinity reagents that target the LIR motif of the SARs optineurin, p62/SQSTM1 and NDP52 (Figure 1A). We started out by analyzing available structures of LC3B and GATE-16 (GABARAPL2) (Paz et al., 2000; Rogov et al., 2013) and identified 19 residues that are in close contact with an overlaid ligand. These residues are distributed over two distinct regions localized on α1 helix (region 1), which interacts with the acidic part flanking the LIR peptides, and on β2 – α2 (region 2), which is in contact with the hydrophobic part of the LIR (Figure 1B). For the construction of phage displayed libraries of LC3B and GATE-16 variants, surface exposed residues in region 1 within 4.5 Å of the peptide ligand and structural overlap in both scaffold proteins were randomized. In region 2, we followed a similar strategy with two exceptions: in LC3B, we randomized the bulky Ile^68^ to allow for structural flexibility in the positioning of the α2 helix, which is in direct contact with the LIR motif. In GATE-16, we chose to randomize Val^51^ to enable the optimization of van der Waals contacts to the conserved aliphatic +3 residue of the LIR motif (Figure 1C). In total, we selected 19 amino acid residues in LC3B and GATE-16 to be mutated by ssDNA-based site directed mutagenesis using a soft randomization strategy (Sidhu et al., 2000; Ernst et al., 2013). Soft randomization means that in each codon triplet encoding the target amino acid, the wt nucleotide occurs with a probability of 70%, while the remaining 30% are evenly distributed among the three non-wt nucleotides. For a library of 19 residues, such an approach results in a mild mutational load of five to six mutations on average, which not only maintains the overall folding of the LC3 proteins but also introduces sufficient surface variations required for a subtle optimization of existing intermolecular contacts (Ernst et al., 2013; Wiechmann et al., 2017, 2020). After mutagenesis, our final combinatorial libraries contained in average 6.5 × 10^9^ unique variants of LC3B or GATE-16 displayed on filamentous phage.

**FIGURE 2:**
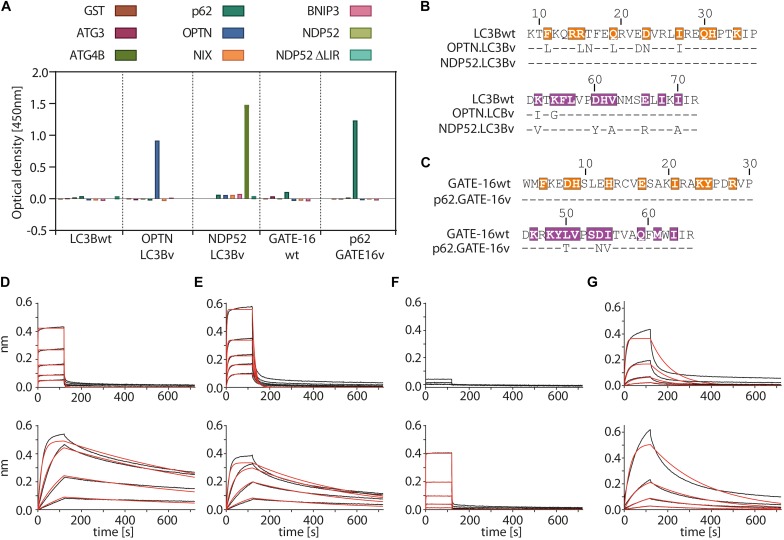
Variants of LC3B and GATE-16 are selective to their cognate LIR and have high affinity *in vitro*. **(A)** Phage ELISA of LC3Bwt and GATE-16wt and selected variants with specificity to the LIR motifs of OPTN, p62 or NDP52. Binding of OPTN.LC3Bv and p62.GATE-16v was tested against ATG3, ATG4B, p62, OPTN, NIX and BNIP3. Variant NDP52.LC3Bv was tested against p62, OPTN, BNIP3, NIX, NDP52 and NPD52 ΔLIR. Background correction to BSA signal was applied. **(B,C)** Sequences of the LC3B and GATE-16 variants with binding to OPTN, NDP52 and p62. Dashes indicate wt residues. Color coding of region 1 and region 2 as in Figure 1C. **(D–G)** Biolayer interferometry (BLI) measurements of LC3Bwt, GATE-16wt (upper panels) and engineered variants (lower panels) against their cognate LIR-peptide. **(C)** LC3Bwt and OPTN.LC3Bv binding to OPTN peptide. **(D)** LC3Bwt and OPTN.LC3Bv binding to pS177 OPTN peptide. **(E)** LC3Bwt and NDP52.LC3Bv binding to NDP52 LIR peptide. **(F)** GATE-16wt and p62.GATE-16v binding to p62 LIR peptide.

In the first selection experiment, we used both phage displayed libraries separately to isolate variants with high affinity to the LIR motifs of OPTN and p62 using phage display. As target proteins we used LIR-containing fragments of OPTN (aa: 2–311) and p62 (aa: 232–370 of isoform 1) fused to GST. These fragments were previously shown to bind to LC3/GABARAP family of proteins in isolation (Pankiv et al., 2007; Wild et al., 2011). For selection of NDP52 binders from the LC3B variant library, we used the atypical cLIR containing peptide (aa: 124–146 of isoform 3) fused to GST as antigen. Here, we were interested in whether the LC3B binding pocket could be modified to recognize an atypical cLIR sequence that was shown to bind only LC3C and had no affinity for either LC3B or GATE-16 (Von Muhlinen et al., 2012). To avoid background binding in the OPTN and p62 selection experiment, non-specific binders were depleted by counter selection on GST. In case of NPD52, counter selection was performed on a GST fusion of a mutated version of the peptide, in which the core LIR sequence was replaced by triple repeat of Ser (called afterward ‘ΔLIR’). After four or five rounds of enrichment on immobilized target proteins, screening of individual clones showed that we isolated variants which bind to all three individual LIR motifs. All selected binders were tested for specificity against a set of unrelated LC3B binding proteins (Figure 2A and Supplementary Figure S1A). In case of OPTN, the selection resulted in three unique binders and based on specificity ELISA data and sequence analysis the variant OPTN.LC3Bv was chosen for further characterization (Figure 2B). In the selection against NDP52, we identified seven unique LC3B variants that showed increased binding *in vitro* (Supplementary Figure S1A). After specificity ELISA, we chose NDP52.LC3Bv as candidate for further characterization (Figure 2B). In addition to the set of unrelated LIR-containing peptides, the variant NDP52.LC3Bv was also tested with the ΔLIR NDP52 GST fusion peptide (Figure 2A). The results indicate that the selected variant binds directly to the core LIR motif of NDP52. The selection against p62 gave 17 binders from LC3B and GATE-16 libraries, indicating that both scaffolds are equally well suited for the selection of optimized binders to LIR motifs. In order to identify the p62 binder with the best binding profile, we carried out specificity ELISA and phage IC_50_ experiments (Supplementary Figures S1A,B). These experiments revealed that some variants isolated in the p62 selection bound to two or more LIR motifs, indicating that they lacked specificity to their cognate peptide *in vitro*. However, six variants were specific to p62 from which one variant, p62.GATE-16v, originating from the GATE-16 library, had the best affinity in phage IC_50_ ELISA (Figure 2C). Importantly, in all aforementioned phage specificity ELISA experiments, the corresponding wt controls of LC3B or GATE-16 displayed on phage did not show any considerable binding to OPTN, p62 or NDP52 under the assay conditions.

In the next step, we asked to what extent the mutations improve the binding of the variants to their target LIR peptides relative to the wt protein by measuring their binding constants. The selected variants and LC3B/GATE-16wt controls were expressed in bacteria, purified as HIS-tag fusion proteins and their affinity to biotinylated target LIR peptides was measured using biolayer interferometry (BLI) (Figures 2D–G and Table 1). In this experiment, the selected variant OPTN.LC3Bv bound the OPTN LIR motif with a *K*_*D*_ of 3.7 nM (Figure 2D). In contrast, the LC3Bwt bound the LIR of OPTN with a *K*_*D*_ of 8.4 μM indicating an over 2000 fold increase in affinity of OPTN.LC3Bv. Additionally, phosphorylation of S177 in the OPTN LIR peptide, which increases the affinity of the LC3Bwt 10-fold to a *K*_*D*_ of 800 nM, does not affect the binding constant of the OPTN.LC3Bv (Figure 2E). In case of NDP52.LC3Bv, we measured a *K*_*D*_ of 1.3 μM for the binding to the NDP52 LIR peptide, while for the LC3Bwt no binding could be detected under the same experimental conditions (Figure 2F). Finally, the variant p62.GATE-16v bound its cognate LIR peptide with a *K*_*D*_ of 62 nM. Unfortunately, a binding constant of the interaction of GATE-16wt with p62 LIR could not be derived because of the heterogeneous binding mode under the conditions tested. However, comparison to a recent report describing an affinity of GATE-16wt to a p62-LIR of 5.2 μM shows that the engineered p62.GATE-16v has a more than 80-fold improved affinity (Wirth et al., 2019). In summary, these results provide evidence that our protein engineering approach yielded variants of human LC3/GABARAP proteins that bind with high affinity to OPTN, NDP52 or p62.

**TABLE 1:** Binding constant *K*_*D*_ of wt controls and engineered variants to their cognate peptides (nM).

Variants	OPTN (172–186)	pS177 OPTN (172–186)	NDP52 (127–141)	p62 (332–346)
LC3Bwt	8,400 ± 1,300	800 ± 48	no binding detected	–
0PTN.LC3Bv	3.7 ± 0.1	4.3 ± 0.1	-	–
NDP52.LC3Bv	–	–	1,300 ± 320	–
GATE-16wt	–	–	–	Heterogenous
p62.GATE-16v	–	–	–	63 ± 1.5

**FIGURE 3:**
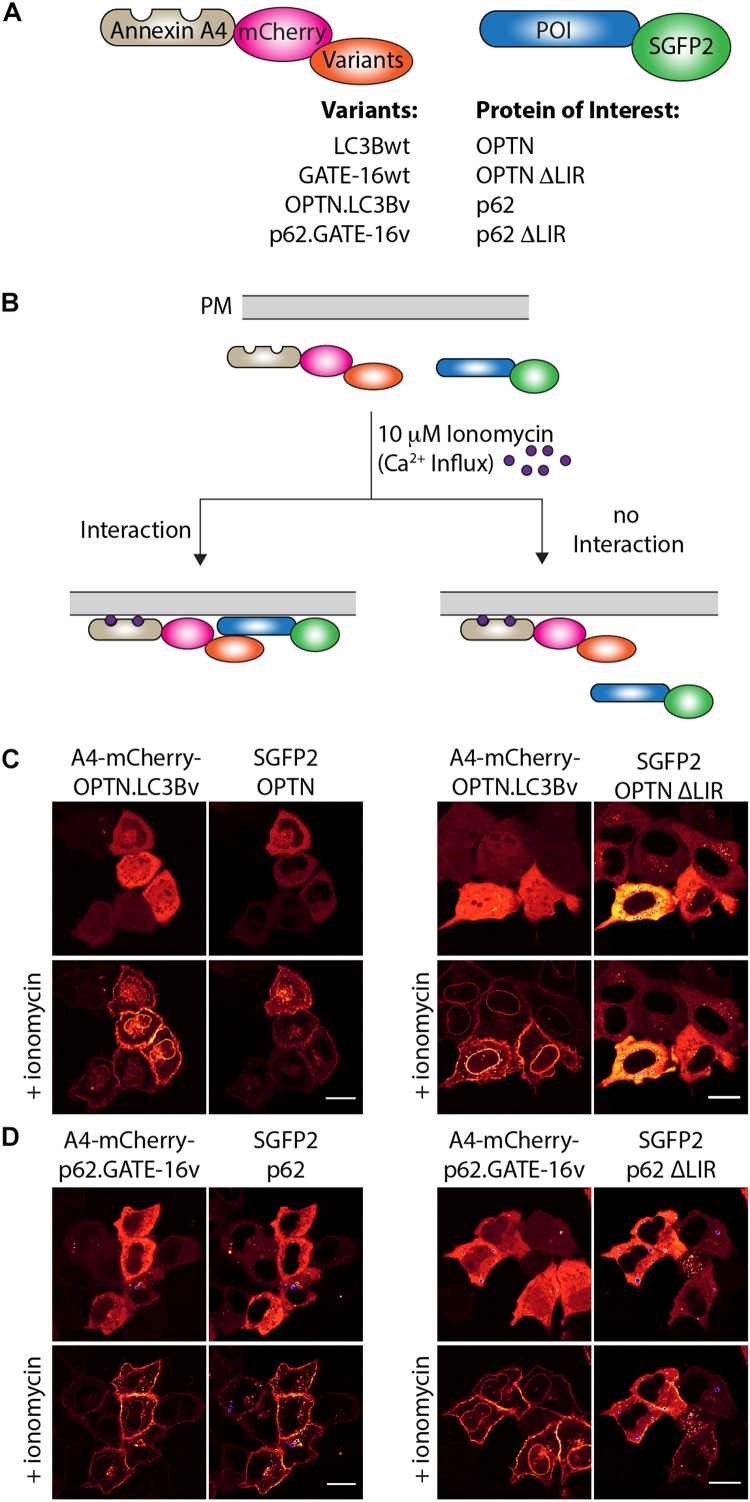
OPTN.LC3Bv and p62.GATE-16v bind their cognate LIR peptide in cells. **(A)** Constructs tested in annexin A4 translocation assays. Annexin A4 (blue) is fused to mCherry (magenta) and the corresponding LC3Bwt, GATE-16wt or variant (orange). Protein of interest (POI) is fused to SGFP2 (green). **(B)** Annexin A4 assay principle. Addition of ionomycin leads to Ca^2+^ influx, resulting in annexin A4 translocation to the plasma membrane. In case the LC3B, GATE-16wt or variant interacts with the POI, green and red fluorescence co-localize at the plasma membrane. In case there is no interaction only the red fluorescence localizes to the plasma membrane. **(C)** Annexin A4 translocation in transfected HeLa cells transfected with OPTN.LC3Bv and SGFP2-OPTN or SGFP2-OPTN ΔLIR upon ionomycin addition. **(D)** Annexin A4 translocation in transfected HELA cells of p62.GATE-16v and SGFP2-p62 or SGFP2-p62 ΔLIR upon ionomycin addition. In **(C)** and **(D)**, bar represents 20 μm.

Next, we were interested in testing if the variants targeting p62 and OPTN bind to their cognate LIR motif in cells. To this end, we used an annexin A4-based membrane co-translocation assay (Piljic and Schultz, 2008), which we previously adapted to test the binding of high affinity LIR-like peptides to LC3/GABARAP proteins (Stolz et al., 2017). We cloned p62.GATE-16v, OPTN.LC3Bv and corresponding wt controls lacking the C-terminal Gly residue to prevent conjugation to PE in frame with annexin A4 (A4) and mCherry, as well as full length OPTN, p62 and corresponding controls in which four core LIR amino acid residues were deleted (ΔLIR), as fusion with enhanced green fluorescent protein (SGFP2) (Figure 3A). Treatment of cells transfected with both constructs with ionomycin results in an intracellular Ca^2+^ influx and subsequent translocation of the annexin A4 fusion protein to the cytosolic side of the plasma membrane as well as to the nuclear envelope. In case the variants interact with their target LIR, a co-localization of the SGFP2 fluorescence with mCherry fluorescence on the cellular membranes can be detected (Figure 3B). As anticipated from our *in vitro* experiments, A4-mCherry-OPTN.LC3Bv construct co-localized with SGFP2-OPTN construct at the plasma membrane after addition of ionomycin (Figure 3C). Importantly, the corresponding SGFP2-OPTN ΔLIR construct did not co-localize at the membranes indicating that the variant is indeed interacting with the LIR motif in the full-length protein. In an additional control experiment, we found that an A4-mCherry-LC3Bwt fusion protein does not co-translocate with SGFP2-OPTN, suggesting that its interaction is not strong enough to mediate binding in this assay (Supplementary Figure S2A). Using the same method, we tested the interaction of the SGFP2-p62 with A4-mCherry-p62.GATE-16v and could observe co-localization at the plasma membrane upon ionomycin addition (Figure 3D). However, we also observed certain degree of co-translocation of the variant construct with the SGFP2-p62 ΔLIR control (Figure 3D). Further controls revealed that the A4-mCherry-GATE-16wt, in addition to interacting with SGFP2-p62, also showed detectable co-translocation with the SGFP2-p62 ΔLIR construct (Supplementary Figure S2B). We suspect that this unexpected translocation of SGFP2-p62 ΔLIR construct is due to the interaction of the A4-mCherry-p62.GATE-16v and GATE-16wt with endogenous p62, which may form oligomers with overexpressed SGFP2-p62 ΔLIR (Ciuffa et al., 2015). Additionally, we tested with this experimental setup if the OPTN.LC3Bv and p62.GATE-16v are cross specific. By co-transfecting SGFP2-OPTN with A4-mCherry-p62.GATE-16v and SGFP2-p62 with A4-mCherry-OPTN.LC3Bv, we were able to demonstrate that the variants lack cross-specificity (Supplementary Figure S2C). Consequently, these results provide evidence that the OPTN.LC3Bv and p62.GATE-16v bind the LIR motif in the full length proteins in living cells and that the variants do not cross react with non-cognate target. NDP52.LC3Bv was not tested in this experiment because *K*_*D*_ of 1.3 μM of its binding affinity is above the threshold for efficient translocation in this assay (Piljic and Schultz, 2008).

**FIGURE 4:**
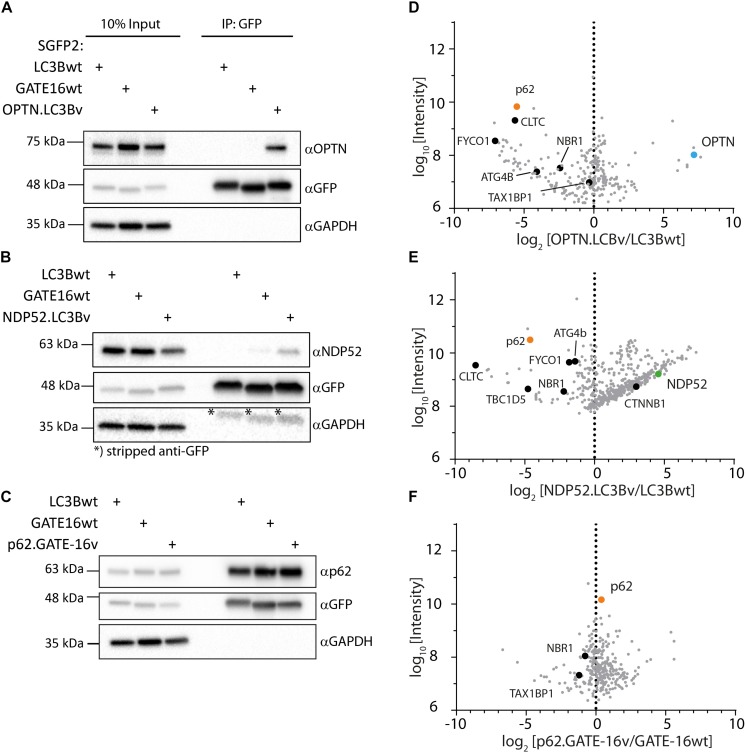
Engineered LC3B and GATE-16 variants co-immunoprecipitate endogenous OPTN, p62 or NDP52. **(A–C)** Co-immunoprecipitation (Co-IP) of **(A)** OPTN, **(B)** NDP52, or **(C)** p62 binding variants and corresponding wt controls expressed as SGFP2 fusion proteins in stable inducible HeLa cells. Western blots were probed with indicated antibodies. **(D–F)** Mass spectrometry of Co-IPs using anti-GFP beads from stable HeLa cells expressing inducibly SGFP2 fusion proteins of LC3Bwt, GATE-16wt, OPTN.LC3Bv or p62.GATE-16v. In case of NDP52.LC3Bv, HeLa cells were transiently transfected with SGFP2 fusion constructs either expressing LC3Bwt or NDP52.LC3Bv. Detected proteins (gray) were plotted as log_2_ enrichment vs log_10_ intensity. Endogenous OPTN (blue), p62 (orange), NDP52 (green) and other LIR containing proteins (black) are shown as indicated.

In order to get a full picture of the intracellular specificity of the engineered variants, we generated stable doxycycline-inducible HeLa Flp-In T-Rex cell lines which expressed SGFP2 fusions of the engineered OPTN.LC3Bv and p62.GATE-16v or corresponding wt controls and performed co-immunoprecipitation experiments combined with mass spectrometry. In case of NDP52.LC3Bv, HeLa cells were transiently transfected with the corresponding SGFP2 construct and used in co-immunoprecipitation mass spectrometry experiment. In our preliminary immunoblot experiments of SGFP2-mediated pull-downs of overexpressed engineered binders, SGFP2-OPTN.LC3Bv showed that the variant efficiently co-immunoprecipitated endogenous OPTN (Figure 4A). Importantly, no OPTN was detected in the corresponding wt controls with GATE-16 or LC3B, demonstrating the greatly improved affinity of the variant compared to wt proteins. Similarly, endogenous NDP52 was co-immunoprecipitated by the NDP52 binding variant, although in this case NDP52 was also immunoprecipitated to a minor extent by GATE-16wt (Figure 4B). It is important to note however, that LC3B, which was the scaffold on which NDP52.LC3Bv was evolved, did not immunoprecipitate any detectable levels of NDP52. In case of the p62.GATE-16v, we also observe a strong enrichment of endogenous p62 (Figure 4C). However, in contrast to the variants that bind OPTN or NDP52, p62 could be also efficiently co-immunoprecipitated by both wt controls, most likely due to the aforementioned oligomerization with endogenous p62. This oligomerization results in a high binding avidity for LC3/GABARAP-wt LIR interactions, which allows an efficient co-immunoprecipitation already by weak interaction partners. Next, we probed the immunoprecipitates from HeLa cells by mass spectrometry (Supplementary Table S1). Detected proteins were compared to a list of known LIR-containing proteins to identify potential off-target interactions partners (Supplementary Table S2) (Wild et al., 2014). As expected from the *in vitro* data, OPTN.LC3Bv efficiently co-immunoprecipitated OPTN, while other known LIR-containing proteins were depleted (Figure 4D). Similarly, to OPTN.LC3Bv, known LIR-containing proteins are reduced in case of NDP52.LC3Bv, but we detected considerably more proteins in the variant co-immunoprecipitation than with LC3Bwt (Figure 4E). GO-term analysis showed that the majority of proteins, which are co-immunoprecipitated in the variant sample are predominantly involved in nucleic acid binding (Supplementary Figure S3). Importantly, the only LIR motif-containing protein that is enriched in addition to the cognate target NDP52 and has been previously shown to interact with LC3/GAPARAPs is β1-catenin (Petherick et al., 2013). It is difficult to explain the observed enrichment of β1-catenin since its putative LIR motif (SHWPLIKAT) and the LIR of NDP52 do not share any sequence similarity. However, a direct interaction of NDP52.LC3Bv with β1-catenin is unlikely, since in such case it would be expected that other LIR-containing proteins would also be enriched due to non-specific binding. In case of the p62-binding GATE-16 variant, enrichment is less pronounced, most probably, again, due to oligomerization of p62 and the resulting avidity effect (Figure 4F). Nevertheless, our data provides evidence that the engineered variants interact with their cognate LIR motif in cells and co-immunoprecipitate their target proteins at endogenous expression levels. Although, in the cases of the p62 binder and the NDP52 binder, the mass spectrometry specificity data is less clear, our *in vitro* data indicates that the constructed LC3B and GATE-16 variants are selective to their target protein. This discrepancy may be due to several factors that influence the mass spectrometry data. It is important to note that the engineered variants are designed to target only a four-amino acid stretch in otherwise large multi-domain proteins, which interact with many other proteins in various cellular compartments that are potentially co-immunoprecipitated. In addition, biophysical properties such as the oligomerization of p62 into larger multimeric assemblies or the relative abundance of the target proteins also may obscure the MS data.

**FIGURE 5:**
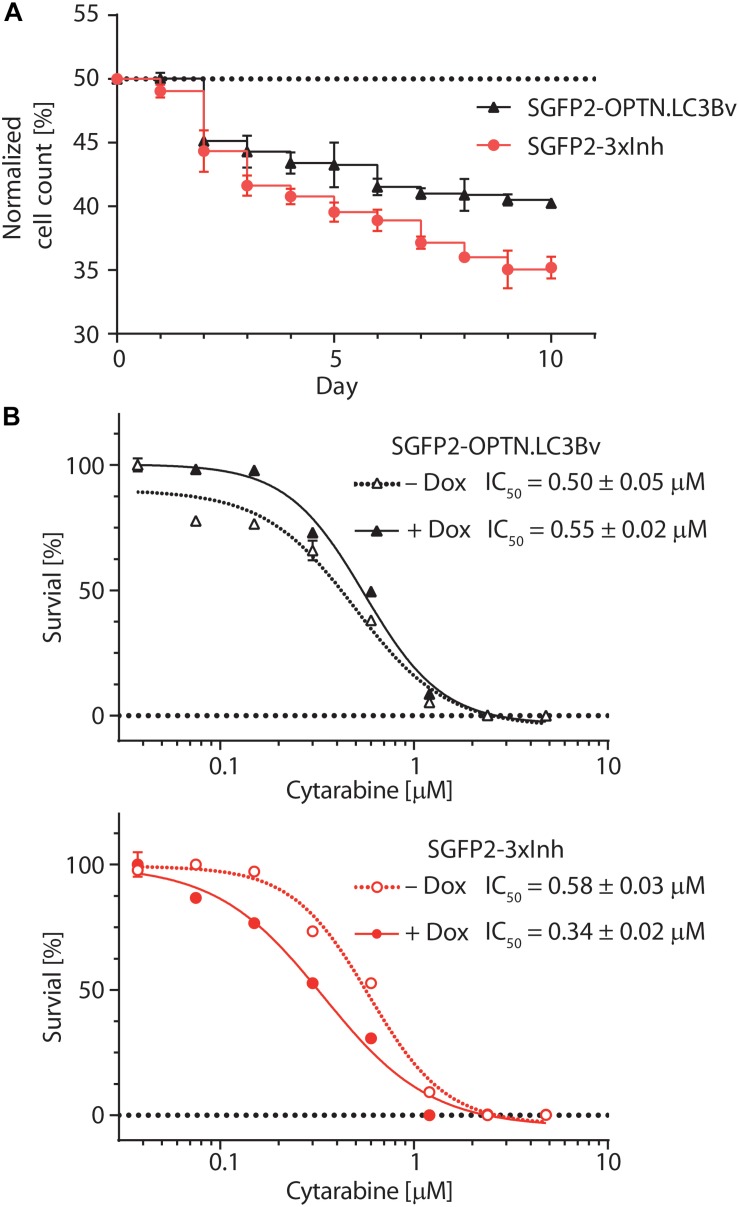
Engineered LC3B and GATE-16 variants reduce growth and sensitize THP-1 AML cells to cytarabine treatment. **(A)** Competitive growth of lentivirally transduced THP-1 cells stably and inducibly expressing SGFP2-OPTN.LC3Bv (black) or SGFP2-OPTN.LC3Bv-p62.GATE-16v-NDP52.LC3Bv (3xInh, red) in relation to similarly transduced THP-1 cells expressing mCherry fusion of LC3Bwt or a triple control construct of LC3Bwt-GATE-16wt-LC3Bwt. Cells transduced with inhibitor construct and corresponding controls were mixed 1:1 and monitored by FACS every 24 h over 10 days. Expression of the transgenes was induced with doxycycline (Dox, 0.8 μg/mL). Percentage of cells expressing SGFP2 inhibitor construct is plotted for each day. Error bars correspond to standard deviation (SD) of three independent replicates (*n* = 3). **(B)** Percent survival of THP-1 cells transduced with sGFP2-OPTNLC3Bv (upper panel) or sGFP2-OPTN.LC3Bv-p62.GATE-16v-NDP52.LC3Bv (3xInh, lower panel) in response to increasing cytarabine concentration in the presence (filled symbols) or absence (open symbols) of doxycycline measured as cellular ATP content by luciferase bioluminescence. Error bars correspond to the SD of three independent replicates (*n* = 3). IC_50_ was determined by non-linear regression using a standard dose response model (GraphPad Prism 7.02).

To test the variants in a cellular model, we asked how the engineered binders affect the growth and fitness of the AML cell line THP-1 (Tsuchiya et al., 1980). Previous work on p62 knock-outs has shown that THP-1 cells are sensitive to the impairment of selective autophagy (Nguyen et al., 2019). To address this question, equal numbers of stably transduced THP-1 cells expressing SGFP2-OPTN.LC3Bv or mCherry-LC3Bwt in a doxycycline-inducible manner were mixed, co-cultured in solution for 10 days in the presence of doxycycline and fractions of the total cell population were monitored daily using a flow cytometer (Figure 5A). This experiment showed that the number of THP-1 cells expressing OPTN.LC3Bv decreased over time, indicating a growth disadvantage compared to LC3Bwt expressing cells. Encouraged by this initial result and the fact that SARs are functionally redundant, we generated a ‘polycistronic’ construct where SGFP2 was fused to OPTN.LC3Bv followed by a T2A site and Myc-p62.GATE16v, and a second T2A site followed by Flag-NDP52.LC3Bv (Kim et al., 2011). This construct encodes all three LC3B and GATE-16 variants (SGFP2-3xInh) simultaneously and expresses the transgene in an inducible fashion in cells (Supplementary Figure S4). Correspondingly, the wt controls were cloned in the same manner except that SGFP2 was exchanged with mCherry (3xWt: mCherry-LC3Bwt/T2A/Myc-GATE-16wt/T2A/Flag-LC3Bwt). In an analogous competitive growth assay, we observed that THP-1 cells expressing the 3xInh responded with an aggravated loss of fitness in comparison to OPTN.LC3Bv and that 3xWt control cells outcompeted the growth of 3xInh cells by a ratio of 2:1 after 10 days (Figure 5A). Next, we asked if inhibiting LIR-mediated interactions sensitizes THP-1 cells to drug treatment by exposing the cells to increasing concentrations of cytarabine, a commonly used chemotherapeutic in AML (Stein and Tallman, 2016). In these assays, drug response was measured by determining cellular ATP content as an indicator of metabolic activity and cell survival in dependence of cytarabine concentration. In order to define a concentration range of cytarabine and the baseline response of the THP-1 cell lines transduced with the inhibitor constructs OPTN.LC3Bv or 3xInh, the cytarabine IC_50_ was first determined without the addition of doxycycline (Figure 5B). In absence of the inhibitory variants, both THP-1 cell lines show similar cytarabine baseline sensitivity with an IC_50_ of ∼0.5 μM. Interestingly, this changes when the expression of inhibitory fusion proteins are induced in the corresponding THP-1 cell lines: while the IC_50_ remains at background levels of 0.5 μM in the presence of OPTN.LC3Bv, expression of the 3xInh fusion protein results in a 40% IC_50_ reduction to 0.3 μM (Figure 5B). Consequently, these results indicate that it is not sufficient to inhibit only LIR mediated interaction of OPTN but it is necessary to inhibit multiple LIRs simultaneously to sensitize THP-1 cells to cytarabine treatment. In summary, our data provides evidence that impairing LIR functions results in an overall loss of fitness, indicating the pro-survival function of selective autophagy in THP-1 cells. Additionally, we could show that blocking LIR interactions introduces a vulnerability in THP-1 cells to a first line chemotherapeutic drug.

## Conclusion

Here, we have generated and characterized in live cells inhibitors of an intrinsically disordered linear peptide motif based on the ubiquitin-like LC3B and GATE-16 proteins by using a protein engineering approach. As target peptides, we have chosen the LIR motifs of the multi-domain SARs OPTN, p62 and NDP52, which recognize ubiquitinated cargo destined for degradation by selective autophagy. Amongst eukaryotes, the interactions of the LC3/GABARAP family of proteins with LIR motifs are highly conserved and have evolved to be in a single/double digit μM range. Since this interaction is relatively weak, we reasoned that it is feasible to develop modified LC3 proteins with improved affinity in relation to the wt interaction by engineering their intramolecular contacts to LIRs. The resulting LC3 variants contain mutations in their binding site that enhance binding to their cognate peptide up to 2000 fold. These improvements of the LIR-binding site are also effective *in situ*, as we could show that the endogenous target proteins are bound in live cells. Using these LC3B and GATE-16 variants in an *in vitro* AML model, it became evident that blocking the LIRs of SAR proteins impairs the growth of THP-1 cells and increases their susceptibility to cytarabine treatment. However, our results identify the oligomerization and functional redundancy of SARs as an impediment for a direct competition with LIR interactions, essentially precluding the inhibition of selective autophagy by a single variant. This result is in agreement with a recent report that described the simultaneous knockout of five autophagy receptors as a prerequisite to completely block PINK/Parkin mediated mitophagy, thereby underlining the high degree of redundancy in the organization of selective autophagy (Lazarou et al., 2015).

Several studies have shown that autophagy is exploited to meet energy needs of fast-growing cells or to counter chemotherapeutic agents and has cytoprotective function in leukemia stem cells (LSCs) and leukemic blast cells. Consequently, interfering with autophagy was shown to be an effective strategy to increase the susceptibility of chronic myeloid leukemia and LSCs cells to chemotherapeutics (Bellodi et al., 2009; Rothe et al., 2014; Baquero et al., 2019). However, in AML the cytoprotective contribution of autophagy is more complex and highly context dependent (Rothe et al., 2019). Nevertheless, recent studies on AML indicate that proteins involved in autophagy are promising drug targets to control proliferative phenotypes. For example, it has been shown that knockdown of key components such as ATG7 and p62, sensitizes AML cell lines for combination treatment with a number of different chemotherapeutic drugs (Bosnjak et al., 2014; Piya et al., 2016). Our results expand on these studies and elucidate that interference with SARs increases the sensitivity of an AML cell line to chemotherapeutic treatment. Moreover, our results identify the LIR binding site of the LC3/GABARAP family as a promising target site to develop small molecule inhibitors to block the binding of autophagy receptor proteins to the autophagosome.

## Materials and Methods

### DNA Constructs

The phagemids were constructed by cloning coding sequences of human LC3B (aa 1–120) or GATE-16 (aa 1–116) in between Flag-tag- and pIII (ΔN1/N2)-coding sequences (Fellouse and Pal, 2005). The following GST fusion constructs applied as targets for phage panning and/or as counter selection reagents or targets for phage specificity and IC_50_ ELISA were used: human ATG3 (aa 2–314, UniProt Q9NT62-1), human ATG4A (aa 1–357, UniProt Q8WYN0-1), human ATG4B (aa 1–357, UniProt Q9Y4P1-1), human p62 (aa 232–370, UniProt Q9Y4P1-1), human OPTN (aa 2–311, UniProt Q96CV9-1), human NIX (BNIP3L, aa 1–137, UniProt O60238-1), human BNIP3 (aa 66–178, UniProt Q12983-1), peptide encompassing aa 124–146 of human NDP52 (UniProt Q13137-1) or it’s ‘ΔLIR’ counterpart (i.e., QFRPENEEDI**SSS**TTQGEVEEIE) as well as two LIR-containing peptides of fungal origin – Pa_6948: TSTVDLLGDDTGVEVGGWEALKPST and Pa_WBD1: GKEDESGSTTEVDDDFELVERVQDALVID, as well as their ‘ΔLIR’ versions (TSTVDLLGDDTGVEVGG**A**EA**A**KPST and GKEDESGSTTEVDDD**A**EL**A**ERVQDALVID, respectively). In each case, the abovementioned coding regions were cloned directly downstream of BamHI site of pGEX-4T-1.

The vectors for bacterial expression of N-terminally HIS-tagged LC3B/GATE-16wt or selected variants were generated by Gateway cloning (Invitrogen) of entry constructs encoding human LC3Bwt, OPTN.LC3Bv or NDP52.LC3Bv (aa 1–119) and GATE-16wt or p62.GATE-16v (aa 1–115) with the destination vector pET53-DEST (Novagen). The entry clones contained a stop codon at the 5′ of the insert sequence. The amino acid linker between HIS-tag and LC3B/GATE-16 was VTSLYKKAGS.

The expression vectors for annexin A4-based membrane co-translocation assay were created by Gateway cloning of the abovementioned entry clones with a destination vector encoding human annexin A4 (aa 1–321, NCBI CCDS 1894.1)-mCherry fusion. The latter vector was constructed by appending annexin A4 ORF upstream of mCherry in the pcDNA3.1/nmCherry-DEST (Invitrogen). The linker between annexin and mCherry had aa sequence GPVAT, while the linker sequence between mCherry and the LC3B/GATE-16 in the final expression construct was GPDPSTNSADITSLYKKAGS. The SGFP2-fusion constructs for the co-translocation assay were also constructed by Gateway cloning, using pcDNA3.1/nSGFP2-DEST destination vector (obtained by replacing mCherry with SGFP2 in the pcDNA3.1/nmCherry-DEST) and the entry clones of human OPTN (aa 1–577, UniProt Q96CV9-1), human p62 (aa 1–440, UniProt Q13501-1) or their ΔLIR mutants (i.e., Δ178-181 and Δ338-341, respectively). In the final expression construct, the linker between SGFP2 and OPTN or p62 was also GPDPSTNSADITSLYKKAGS.

The vectors for generation of stable HeLa Flp-In T-REx cells were obtained by Gateway cloning of the abovementioned LC3B/GATE-16 (wt or variant) or an analogous NDP52.LC3Bv entry clones into pcDNA5/FRT/TO/nSGFP2-DEST. The latter was obtained by replacing DNA sequence encoding N-terminal triple Flag in pcDNA5/FRT/TO/n3xFlag-DEST (Invitrogen) by SGFP2 gene. Again, the linker connecting SGFP2 and LC3B/GATE-16 was GPDPSTNSADITSLYKKAGS.

The vectors used for lentiviral transduction and subsequent selection of stable inducible THP-1 cell lines were based on pLD-T/nFlag-DEST destination vector, a gift from Jason Moffat (University of Toronto, The Donnelly Centre). This vector encodes a transcriptional T2A fusion of puromycin resistance marker with rtTA (Tet-responsive reverse transactivator), enabling doxycycline-inducible expression of encoded transgenes. The original destination vector was modified by replacing Flag-tag with either mCherry or SGFP2. To obtain lentiviral constructs for expression of mCherry-LC3Bwt and SGFP2-OPTN.LC3Bv, the respective destination vectors and the abovementioned entry clones were used for Gateway cloning. Also in these cases, the linker between the fluorescent protein and LC3B was GPDPSTNSADITSLYKKAGS. For the assembly of mCherry-3xWt and SGFP2-3xInh constructs, the respective pLD-T destination vectors were reacted with Gateway entry clones encoding fusion construct: LC3Bwt (aa 1–119)/T2A (P**GSGEGR GSLLTCGDVEENPGP**)/Myc (**EQKLISEEDL**GSGS)-GATE-16wt (aa 1–115)/T2A (P**GSGEGRGSLLTCGDVEENPGP**)/Flag (**DYKDDDDK**GSGS)-LC3Bwt (aa 1–119) or its counterpart encoding triple fusion of the selected variants: OPTN.LC3Bv/T2A/Myc-p62.GATE-16v/T2A/Flag-NDP52.LC3 Bv. The inserts for the creation of these entry clones were generated by gene synthesis.

### Construction of the Libraries of LC3B and GATE-16 Variants, Selections of the LC3B/GATE-16 Variants and Their Characterization by IC50 Phage ELISA

M13 phage display libraries of human LC3B and GATE-16 variants were constructed as described before using soft randomization strategy (Fellouse and Sidhu, 2007; Ernst et al., 2013; Wiechmann et al., 2017). The libraries were used for solid phase phage panning experiments and the selection stringency during the subsequent panning steps was increased by extending the number of washing cycles, shifting the incubation/washing from 4°C to room temperature, decreasing the amounts of coated target proteins and increasing the present amounts of the counter selection proteins. Individual clones were analyzed by phage colony and, subsequently, phage specificity ELISA and DNA sequencing after three to five rounds of selection. Phage IC_50_ ELISA was performed as described previously (Wiechmann et al., 2017) but using 1 μM, 500 nM, 250 nM, and 125 nM concentrations of the free binding protein in solution.

### Expression and Purification of GST- and HIS-Tagged Proteins

Protein expression in *E. coli* and their subsequent purification was performed using standard methods, as already described (Wiechmann et al., 2017). The exceptions were using treatment with lysozyme (1 mg/mL)/DNaseI (5 μg/mL)/TRITON X-100 (0.5% v/v) instead of sonication for cell lysis, omission of DTT in the dialysis buffer and protein quantitation using BCA assay kit (Pierce) instead of measuring absorbance at 280 nm. Purified and dialyzed proteins were aliquoted and stored at −80°C for future use.

### Bio-Layer Interferometry (BLI)

Kinetic binding assays were performed on Octet RED96 system (ForteBio) using streptavidin (SA) biosensors (ForteBio). N-terminally biotinylated peptides were synthesized by GenScript at over 75% purity: OPTN GSSEDSFVEIRMAEG, pS177 OPTN GSSED(pS)FVEIRMAEG, p62 SGGDDDWTHLSSKEV and NDP52 PENEEDILVVT TQGE. The running buffer consisted of 150 mM NaCl, 1% (w/v) BSA and (v/v) 0.02% Tween 20 in DPBS without calcium and magnesium (from 10x stock, Gibco). SA sensors loaded with the biotinylated peptide were quenched in 10 μg/mL biocytin. Sensors were dipped into serial dilutions of purified HIS-tagged LC3B/GATE-16 proteins: 5 μM, 1.67 μM, 0.56 μM, 185 nM, 61.7 nM, 20.6 nM and 6.8 nM with the exception of the assay for LC3Bwt binding to NDP52 peptide, where serial dilutions were 20 μM, 10 μM, 5 μM, 2.5 μM, 1.25 μM, 625 nM, 312.5 nM. In each experiment, background binding of peptide-loaded sensor incubated in pure running buffer was recorded and used for background correction. To optimize the χ^2^ and *R*^2^ values of the fit, the sensograms at either the highest or the lowest analyte concentrations were removed and the 1:1 global fitting model was used to determine *k*_*on*_, *k*_*off*_, and *K*_*D*_ values.

### Annexin A4-Based Co-translocation Assay and Microscopy

HeLa Kyoto cells (Carsten Schultz, EMBL Heidelberg), were routinely grown in DMEM high glucose medium (Gibco) supplemented with 10% FCS (Gibco) and 100 units/mL of each penicillin and streptomycin (Gibco) at 37°C in a humidified incubator with 5% CO_2_ in air. One day prior to transfection, cells were trypsinized and seeded in glass bottom dishes (MatTek Corporation). Cells were transiently co-transfected with the A4-mCherry-LC3B/GATE-16 and SGFP2-OPTN/p62 constructs using GeneJuice transfection reagent (Merck) according to manufacturer’s protocol. 24 h post transfection, the medium was changed to DMEM buffered with HEPES and devoid of phenol red (Gibco) and the co-translocation assay was performed as described before (Piljic and Schultz, 2008) on TCP SP8 laser−scanning microscope (Leica) using HC PL APO CS2 63x/1.40 oil−immersion lens. Briefly, images of mCherry fluorescence (excitation 552 nm, emission 562 nm–650 nm) and SGFP2 fluorescence (excitation 488 nm, emission 495 nm–550 nm) were recorded before and shortly after treating the cells with ionomycin at 10 μM end concentration. The images were captured in 8-bit mode, at 400 Hz scanning speed, 512×512 pixel resolution, with bidirectional scanning and with four line averages. The pinhole was opened to 1 Airy unit. For easy discrimination of saturated pixels, the images are presented with Glow (O&U) lookup table – saturated pixels are shown in blue.

### Immunoprecipitation and Immunoblotting

Stable, doxycycline-inducible HeLa Flp-In T-REx cell lines expressing LC3B/GATE-16 proteins were generated using pcDNA5/FRT/TO Gateway expression clones according to the instructions of the provider of the Flp-In T-REx system (Invitrogen). Cells which survived double antibiotic selection (15 μg/mL blasticidin, 300 μg/mL hygromycin) were pooled and routinely cultivated as described above for HeLa Kyoto cell, but in the constant presence of blasticidin and hygromycin. For immunoprecipitation and immunoblotting experiments, 8 × 10^5^ cells were seeded in 5 cm diameter dish, treated with 1 μg/mL doxycycline 24 h after plating and cultivated for further 24 h before cells were rinsed in PBS and lysed in 350 μL lysis buffer [10 mM TRIS pH 7.5, 150 nM NaCl, 0.5% (v/v) ND-40, 1 mM EDTA, protease inhibitor coctail (Roche)] per dish. Lysates were cleared by centrifugation and incubated with GFP-Trap agarose beads (Chromotek). Beads were washed in lysis buffer in which the NP-40 concentration was decreased to 0.2% (v/v) and boiled 5 min at 95°C with reducing sample buffer. After SDS-PAGE and immunoblotting, PVDF membranes were blocked with 5% milk powder in TBST, incubated over night with primary antibody solution in 5% milk at 4°C and probed with secondary antibody-HRP conjugates. Blots were visualized using ECL substrate (Pierce) using ChemiDoc Imager (Bio-Rad). Before probing with consecutive primary antibodies, membranes were stripped in 1.5% (w/v) glycine pH 2.2, 0.1% (w/v) SDS and 1% (v/v) Tween 20. Primary antibodies used: anti-OPTN (Abcam, ab23666), anti-p62 (MBL, M162-3), anti-NDP52 (CST, 60732), anti-GFP (Santa Cruz, sc-9996), anti-GAPDH (CST, 2118).

### Mass Spectrometry

For OPTN and p62 binder experiments, proteins were eluted from the GFP Trap beads with Laemmli buffer and separated by 1D PAGE, each gel lane was cut in four pieces and subjected to in-gel trypsin digestion according to Grumati et al. (2017). For NDP52.LC3Bv, proteins were digested with trypsin on beads and peptides were desalted by StageTip cleanup and analyzed by LC-MS/MS. In brief, the peptides were separated by a non-linear 46 min gradient on an self-packed 20 cm C18 column with an Easy nLC 2 or 1200 (Thermo Fisher) and injected online in an Orbitrap Elite or Q Exactive HF mass spectrometer (Thermo Fisher operating in Top20 or Top15 data dependent mode). For protein identification and LFQ quantification, spectra were extracted and searched against the UniProt Human SwissProt database with MaxQuant 1.6.1. Proteins that were differentially enriched by the specific binder and the corresponding wt were detected by a 5% FDR corrected two sample *t*-test with Perseus 1.6.1. For visualization, log_10_ of peptide intensity was plotted versus log_2_ of fold enrichment relative to the wt sample.

### Lentiviral Transduction and Competitive Growth Assays

Virus preparation was made in HEK293T cells using psPAX and pMD constructs, viral supernatant was collected 72 h post transfection. THP-1 cells were transduced with freshly prepared virus for 24 h and further selected for 5 days with puromycin (1 μg/mL). After induction with doxycycline (0.8 μg/mL) for 24 h, GFP- or mCherry-expressing cells were FACS-sorted and used for competitive proliferation assay following established protocols (Nguyen et al., 2019). In brief, GFP- and mCherry-expressing control cells were mixed in a 1:1 ratio and co-cultured in the presence of doxycycline (0.8 μg/mL). GFP- and mCherry signal in mixed population were monitored daily for 10 days by flow cytometry.

### Cytarabine Titration of THP-1 Cells

THP-1 cells transduced with SGFP2-OPTN.LC3Bv, SGFP2-3xInh, mCherry-LC3Bwt or mCherry-3xWt controls were seeded at 1 × 10^6^ cells per mL and cultured overnight in the presence (+dox) or absence (−dox) of doxycycline. Cells were washed once with PBS and resuspended in the same volume of pre-warmed freshly prepared growth-medium while maintaining the same growth conditions as in the overnight culture. Cell density was adjusted to 5.5 × 10^4^ cells/ml and 90 μL added to individual wells in a 96-well cell culture plate. 10 μl of a cytarabine 1:1 dilution series was added to 90 μl of cells to the indicated final concentrations and incubated for 72 h (37°C/5% CO_2_). Experiment was carried out in three independent replicates and cell viability was measured using CellTiter-Glo (Promega) following the manufactures protocol. Data was analyzed using Graphpad Prism 7.02.

### qPCR

From THP-1 cells transduced with either construct, total RNA was isolated using NucleoSpin RNA Plus (Macherey-Nagel) according to manufacturer’s protocol. 3 μg of RNA were DNAseI treated for 30 min at 37°C. DNAseI was inactivated with 25 mM EDTA for 10 min at 65°C. 1 μg RNA was incubated for 5 min at 70°C with 5 μM oligo-dT and 5 μM random primer followed by 60 min at 42°C for synthesis of cDNA. For quantitative PCR reaction, primer design was optimized to yield 90% efficiency or better and product size was limited to less than 150 bp. PCR was performed in the presence of SYBR Green on a DNA Engine OPTICON machine from MJ Research according to standard protocols. Data were analyzed using OPTICON 2 Software and C(t) was plotted (Supplementary Figure S4). Student *t*-test was performed on four independent replicates with Graphpad Prism 7.02.

## Data Availability Statement

All datasets generated for this study are included in the article/Supplementary Material.

## Author Contributions

MP, ID, and AE designed the project. AE and MP wrote the manuscript. MP, SG, and AV characterized binders *in vitro* and in cells. AE, MP, FB, and CB analyzed the data. OV performed AML cell line experiments. FB performed mass spectrometry and mass spectrometry analysis. All authors commented on the final draft of the manuscript.

## Conflict of Interest

The authors declare that the research was conducted in the absence of any commercial or financial relationships that could be construed as a potential conflict of interest.

## Supplementary Material

FIGURE S1 Selection of specific binders. **(A)** Phage specificity ELISA for p62, OPTN and NDP52 binders obtained after the final round of phage selections. Binding of clones from p62 and OPTN selections was tested with BSA, GST and a panel of GST-fused proteins: ATG4A, ATG4B, ATG3, p62, NBR1, OPTN, BNIP3, and NIX. Binding of clones from NDP52 selections was tested with BSA, GST and GST-fusions of LIR-containing peptides of human NDP52 and two unrelated proteins of fungal origin (Pa6948 and PaWBD1), as well as their ΔLIR versions. In all cases, anti-Flag antibody was used as positive control. **(B)** Phage IC_50_ ELISA of p62-specific clones. Selected clones were pre-incubated in solution with indicated concentrations of GST-p62 and probed on immobilized GST-p62. Results are shown as normalized OD450 to the signal in the absence of competitor.

FIGURE S2 Annexin A4-based membrane co-translocation assay in HeLa cells. Micrographs before (upper row) and after addition of 10 μM ionomycin (lower row) are shown. **(A)** Assay results for LC3Bwt and OPTN. **(B)** Results for GATE-16wt and p62. **(C)** Cross-specificity test for binding of p62.GATE-16v to OPTN (left panel) and of OPTN.LC3Bv to p62. Scale bars represent 20 μm.

FIGURE S3 Gene-ontology analysis of proteins detected in Co-IP MS/MS experiment using SGFP2-NDP52.LC3Bv. Proteins with an enrichment factor of log_2_> 2 relative to LC3Bwt were analyzed using Panther14.1. From 210 proteins submitted, a GO-term enrichment for 127 proteins for the indicated protein function was detected.

FIGURE S4 qPCR analysis of the mRNA transcription of 3xWT or 3xInh constructs. The T2A linked expression cassettes for SGFP2-3xInh or mCherry-3xWT constructs were analyzed using individual primer pairs for each LC3/GABARAP wt or variant construct and GAPDH control. Average amplicon length was 138 bp. The C(t) values of four independent amplification experiments are represented as box plot for each expression cassette in absence (black) or presence of doxycycline (magenta). Error bars correspond to standard deviation of four independent replicates. *P*-values were calculated using an unpaired Students *t*-test using GraphPad Prism 7.02 (ns *p* > 0.05, ^∗∗∗^*p* < 0.005).

TABLE S1 Complete datasets from co-immunoprecipitation and mass spectrometry used to generate Christmas tree plots in Figures 4D–F. Enrichment of OPTN.LC3Bv vs LC3Bwt detected peptides. Enrichment of NDP52.LC3Bv vs LC3Bwt detected peptides. Enrichment of p62.GATE-16v vs GATE-16wt detected peptides.

TABLE S2 List of 24 known LIR containing proteins that have been shown to interact with LC3/GABARAP family (Wild et al., 2014).
