# Supplementary figures and images for: Disrupting the LC3 Interaction Region (LIR) Binding of Selective Autophagy Receptors Sensitizes AML Cell Lines to Cytarabine
Source: Front Cell Dev Biol. 2020 Mar 31;8:208. doi: 10.3389/fcell.2020.00208 (PMC7137635; doi:10.3389/fcell.2020.00208)

Figure S1

A

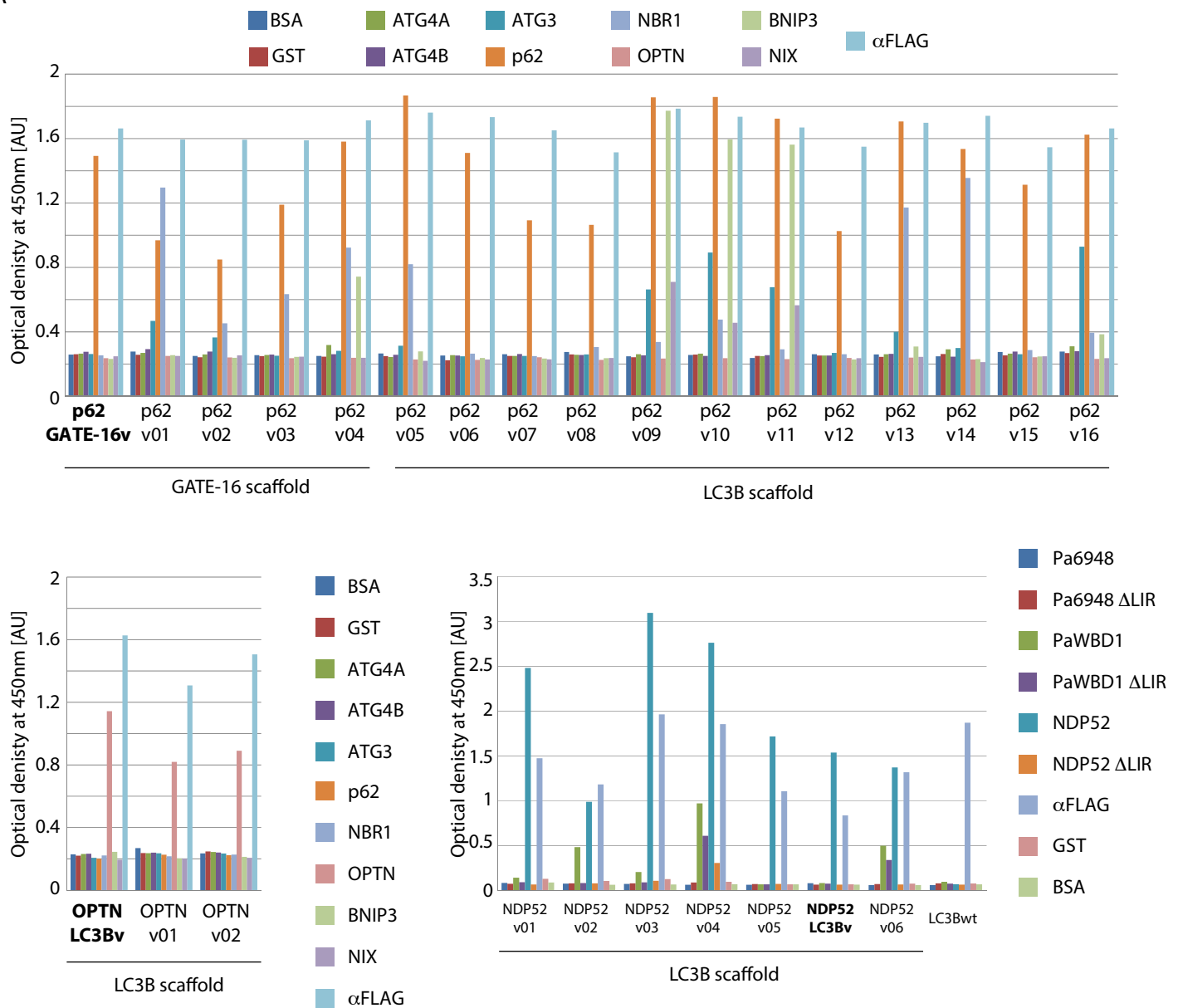

B

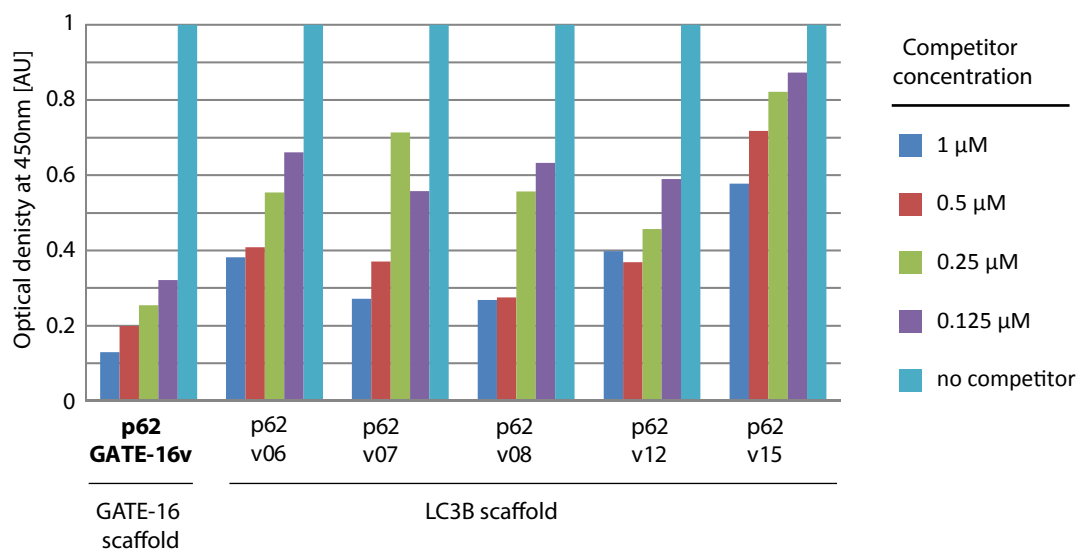

Supplement: FIGURE S1 — Selection of specific binders. (A) Phage specificity ELISA for p62, OPTN and NDP52 binders obtained after the final round of phage selections. Binding of clones from p62 and OPTN selections was tested with BSA, GST and a panel of GST-fused proteins: ATG4A, ATG4B, ATG3, p62, NBR1, OPTN, BNIP3, and NIX. Binding of clones from NDP52 selections was tested with BSA, GST and GST-fusions of LIR-containing peptides of human NDP52 and two unrelated proteins of fungal origin (Pa6948 and PaWBD1), as well as their ΔLIR versions. In all cases, anti-Flag antibody was used as positive control. (B) Phage IC50 ELISA of p62-specific clones. Selected clones were pre-incubated in solution with indicated concentrations of GST-p62 and probed on immobilized GST-p62. Results are shown as normalized OD450 to the signal in the absence of competitor. [file Image_1.pdf]

Figure S2

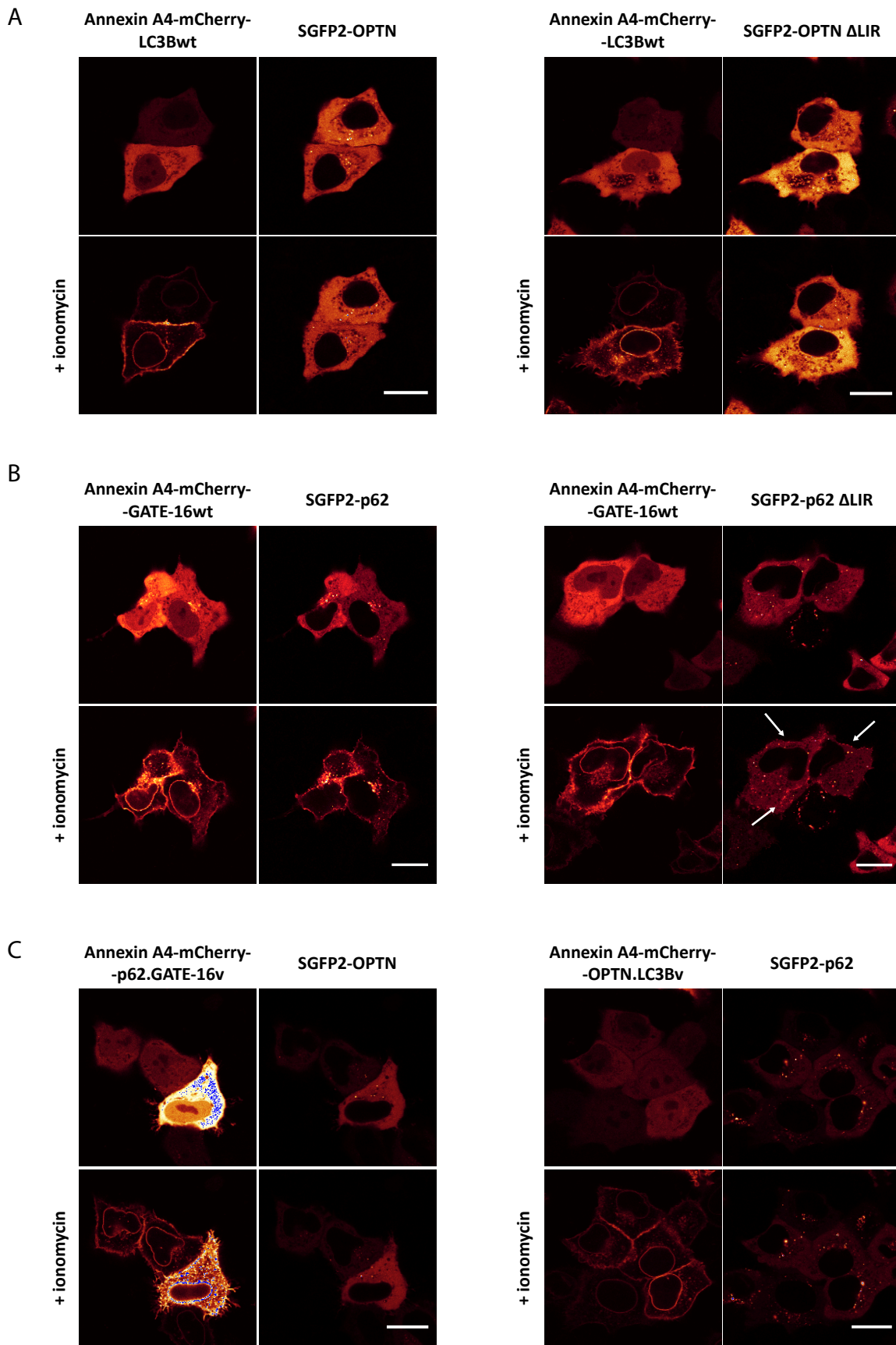

Supplement: FIGURE S2 — Annexin A4-based membrane co-translocation assay in HeLa cells. Micrographs before (upper row) and after addition of 10 μM ionomycin (lower row) are shown. (A) Assay results for LC3Bwt and OPTN. (B) Results for GATE-16wt and p62. (C) Cross-specificity test for binding of p62.GATE-16v to OPTN (left panel) and of OPTN.LC3Bv to p62. Scale bars represent 20 μm. [file Image_2.pdf]

Figure S3

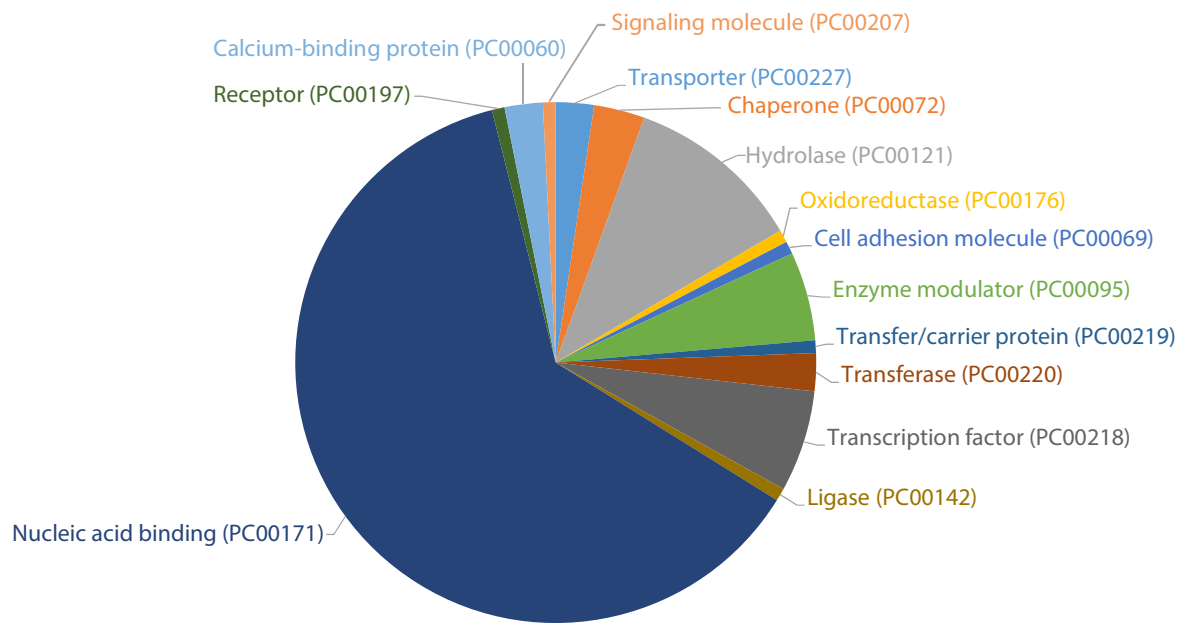

Supplement: FIGURE S3 — Gene-ontology analysis of proteins detected in Co-IP MS/MS experiment using SGFP2-NDP52.LC3Bv. Proteins with an enrichment factor of log2> 2 relative to LC3Bwt were analyzed using Panther14.1. From 210 proteins submitted, a GO-term enrichment for 127 proteins for the indicated protein function was detected. [file Image_3.pdf]

Figure S4

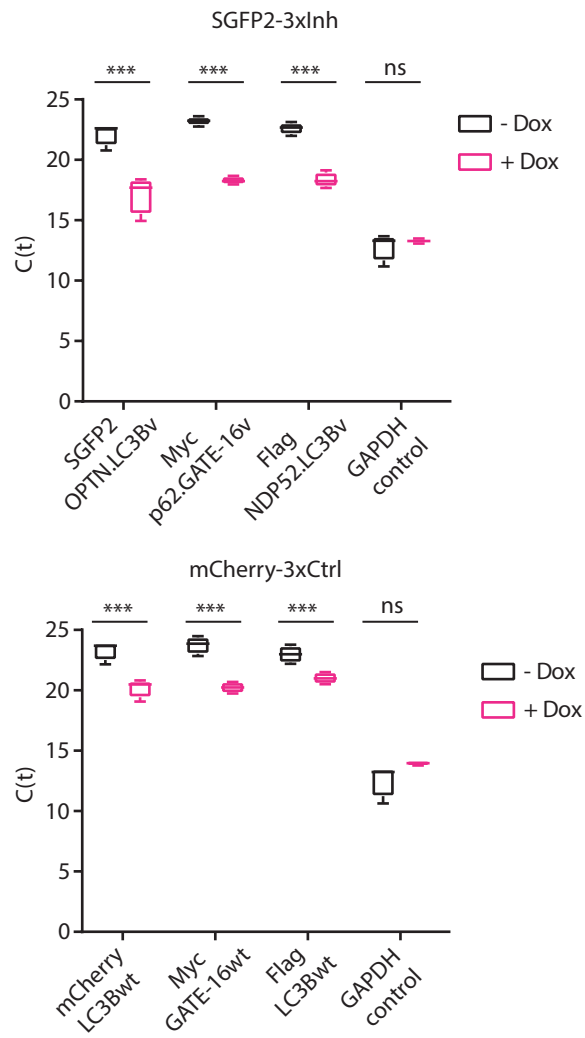

Supplement: FIGURE S4 — qPCR analysis of the mRNA transcription of 3xWT or 3xInh constructs. The T2A linked expression cassettes for SGFP2-3xInh or mCherry-3xWT constructs were analyzed using individual primer pairs for each LC3/GABARAP wt or variant construct and GAPDH control. Average amplicon length was 138 bp. The C(t) values of four independent amplification experiments are represented as box plot for each expression cassette in absence (black) or presence of doxycycline (magenta). Error bars correspond to standard deviation of four independent replicates. P-values were calculated using an unpaired Students t-test using GraphPad Prism 7.02 (ns p > 0.05, ∗∗∗p < 0.005). [file Image_4.pdf]
