# Supplementary material for: Disrupting the LC3 Interaction Region (LIR) Binding of Selective Autophagy Receptors Sensitizes AML Cell Lines to Cytarabine
Source: Front Cell Dev Biol. 2020 Mar 31;8:208. doi: 10.3389/fcell.2020.00208 (PMC7137635; doi:10.3389/fcell.2020.00208)
Supplement: TABLE S2 — List of 24 known LIR containing proteins that have been shown to interact with LC3/GABARAP family (Wild et al., 2014). [file Table_2.pdf]

Table S2:

Uniprot ID	Full name	Gene Name
Q9Y4P1	Cysteine protease ATG4B	ATG4b
O75143	Autophagy-related protein 13	ATG13
O60238	BCL2/adenovirus E1B 19 kDa protein-interacting protein 3-like	BNIP3L
P35222	Catenin beta-1	CTNNB1
P22681	E3 ubiquitin-protein ligase CBL	Cbl
Q96L12	Calreticulin-3	CALR3
Q00610	Clathrin heavy chain 1	CLTC
O14641	Segment polarity protein dishevelled homolog DVL-2	DVL2
Q8IVP5	FUN14 domain-containing protein 1	FUNDC1
Q9BQS8	FYVE and coiled-coil domain-containing protein 1	FYCO1
Q8TD08	Mitogen-activated protein kinase 15	MAPK15
Q14596	Next to BRCA1 gene 1 protein	NBR1
Q13137	Antigen nuclear dot 52 kDa protein	NDP52
Q96CV9	Optineurin	OPTN
Q13501	Sequestosome-1	SQSTM1
Q8TDY2	RB1-inducible coiled-coil protein 1	RB1CC1
O95210	Starch-binding domain-containing protein 1	STBD1
Q86VP1	Tax1-binding protein 1	TAX1BP1
Q92609	TBC1 domain family member 5	TBC1D5
Q3MII6	TBC1 domain family member 25	TBC1D25
Q96A56	Tumor protein p53-inducible nuclear protein 1	TP53INP1
Q8IXH6	Tumor protein p53-inducible nuclear protein 2	TP53INP2
O75385	Serine/threonine-protein kinase ULK1	ULK1
